# A Case of Need: Linking Traits to Genebank Accessions

**DOI:** 10.1089/bio.2018.0033

**Published:** 2018-10-12

**Authors:** Noelle L. Anglin, Ahmed Amri, Zakaria Kehel, Dave Ellis

**Affiliations:** ^1^ CIP-International Potato Center, Lima, Peru.; ^2^ ICARDA-International Center for Agricultural Research in the Dry Areas, Rabat, Morocco.

**Keywords:** genetic resources, trait association, genebanks, FIGS, molecular markers, GWAS

## Abstract

Genebanks are responsible for collecting, maintaining, characterizing, documenting, and distributing plant genetic resources for research, education, and breeding purposes. The rationale for requests of plant materials varies highly from areas of anthropology, social science, small-holder farmers, the commercial sector, rehabilitation of degraded systems, all the way to crop improvement and basic research. Matching “the right” accessions to a particular request is not always a straightforward process especially when genetic resource collections are large and the user does not already know which accession or even which species they want to study. Some requestors have limited knowledge of the crop; therefore, they do not know where to begin and thus, initiate the search by consultation with crop curators to help direct their request to the most suitable germplasm. One way to enhance the use of genebank material and aid in the selection of genetic resources is to have thoroughly cataloged agronomic, biochemical, genomic, and other traits linked to genebank accessions. In general, traits of importance to most users include genotypes that thrive under various biotic and abiotic stresses, morphological traits (color, shape, size of fruits), plant architecture, disease resistance, nutrient content, yield, and crop specific quality traits. In this review, we discuss methods for linking traits to genebank accessions, examples of linked traits, and some of the complexities involved, while reinforcing why it is critical to have well characterized accessions with clear trait data publicly available.

## Introduction

*E**x situ* collections such as genebanks serve to maintain genetic diversity for current and future use in crop improvement, research, and educational programs. Maintenance of genetic resources into perpetuity along with making this material available to researchers worldwide helps ensure food security for the future. The underlying genetic diversity in the plant germplasm is the lifeblood of plant breeding, making conservation of the diversity of major crops critical, and mining these collections for useful traits.^1^ However, the major obstacle to enhancing genebank materials is the lack of adequate evaluation data, and thus, the inability to adequately respond to inquiries for those particular accessions that directly meet the needs of the user.^2^ For the majority of germplasm accessions, only basic passport data (an internationally accepted set of data that genebankers use to provide minimum necessary information about the accessions in their collections, www.genbank.at/en/national-inventory/database-descriptors/passport-data.html) is available and data on unique proprieties/traits is generally lacking.^3,4^ Even newly acquired material rarely has more information associated with it other than basic passport information. While passport information is important, it often does not help a user of the genebank to discern which of the thousands of accessions in a database potentially contain the trait they want.

Collecting trait data is labor intensive, costly, and requires multiple sites/years to evaluate the magnitude and structure of genotype-by-environment (GxE) variation in the expression of a given trait that is influenced by the population and the environments under study. Environments should cover a wide range of geographical locations and seasons for better decision making on which accession performs optimally in varying environments. A minimum of three locations are needed to evaluate GxE for most agronomic traits, but more locations may be needed to fully predict how the trait is influenced by the environment. Even when extensive phenotyping is performed with multiple locations evaluated, it is often difficult to display the information succinctly in a database (dbase) or predict how the accession would perform in a new environment.

In the Second Report on The State of the World's Plant Genetic Resources for Food and Agriculture,^5^ it was estimated that there are over 1750 genebanks worldwide, holding ∼7.4 million accessions; yet, the report claims that only 25%–30% of these accessions are genetically unique. Furthermore, the majority of these collections are securely preserved, but largely unused. Even though *ex situ* collections have increased over the last few decades in a global effort to conserve plant genetic resources, the size of these collections complicate the maintenance and evaluation of these genetic resources.^6^ Moreover, the lack of publicly available information has resulted in low use of these resources.^7^

Historically, many of the accessions added to genebank collections came without information on specific traits or sometimes even lacking basic passport information. In addition, some genetic resources were collected by eager scientists on mission trips to find new species or explore new geographical locations for their crop of interest and sometimes only basic information such as the collecting site or putative species was noted due to time constraints of the mission. Also, older plant collection trips did not always have easy access to GPS locators or GPS applications on cellular phones. In other cases, material was collected in open air markets or donated from other collaborators and arrived in the genebank without any information on its parentage or unique attributes. Evaluating, accurately scoring, and documenting most traits of agronomic, nutritional, or other interest often requires several years of intensive field phenotyping and/or specialized equipment for laboratory measurements along with bona fide documentation so the information becomes available to the community.

Genebanks are often referred to as the crown jewels in organizations to which they belong. They house numerous plants that have been collected worldwide and preserved *ex situ* for future generations to utilize. The plants and seeds maintained in these genebanks hold various genes and traits that may be the keys to solving current or future biotic and abiotic challenges that arise, especially important considering the pressures from a changing climate. Genetic resources, however, are often “diamonds in the rough” and need further work, such as extensive evaluation to uncover their true nature or prebreeding efforts, to elucidate their value. The value of some accessions may never be realized especially if they remain uncharacterized and unutilized like many of the holdings in global genebank.

A great example of a “diamond in the rough” is PI 203396 (*Arachis hypogaea*), which was collected in 1952 in a market in Porto Alegre, Brazil and added as an accession to the peanut germplasm collection in the United States Department of Agriculture (USDA) genebank in Griffin, GA USA. PI 203396 sat mainly unused except for regular regeneration cycles needed to keep the seed viable. Yet, this accession contained gene(s) for resistance to tomato spotted wilt virus (TSWV) resistance and the incorporation of these alleles into peanut varieties has now been estimated to have an economic value of more than $200 million annually.^8^ Even today, the majority of the peanut varieties released have some ancestry linked back to PI 203396 or its derivatives to confer TSWV resistance. This diamond in the rough likely would have never been discovered if it hadn't been for TSWV devastating the peanut production in the Southeast of the United States starting in 1987 with little to no resistance in commercially grown varieties at that time. Breeders raced to find a solution and it came from a single accession. Even though it is well known that this accession confers TSWV resistance and its alleles are integrated into most of the commercial peanut varieties grown today, the information is not documented or associated with this accession in the observational data in the USDA Genetic Resources Information Network (GRIN) public dbase. This makes it hard to impossible for worldwide researchers or those new to peanut breeding to find information and order this accession that contains TSWV resistance. Notably, in GRIN this accession is listed as being resistant to leaf spot.

The peanut accession PI 203396 is not a unique case of undocumented material in genebanks. In the Second Report on the state of the world's plant genetic resources for food and agriculture,^5^ they state that there are considerable gaps in basic documentation and characterization data for plant genetic resources, and this is a major limitation for use of Plant and Genetic Resources for Food and Agriculture (PGRFA) in breeding programs. Many *ex situ* banks are still documenting information on paper or Excel sheets and do not have a publicly accessible dbase. As a whole, genebanks have characterized and evaluated their collections, but on average only 64% are characterized morphologically, 51% agronomically, 14% biochemically, 14% for abiotic traits, and 22% for biotic traits. One of the main recommendations from this report^5^ was to strengthen the characterization and evaluation of these genetic resources to encourage and increase the use of germplasm. Further, search tools and public dbases are needed to hold and make accessible all the information. Even in well funded genebanks with public dbases significant gaps appear for various traits where some accessions will have data and the rest are uncharacterized. For example, the International Potato Center (CIP) has over 5800 accessions with morphological or trait data for potato, but the number of accessions characterized for each trait vary from 2 to over 5000 accessions with morphological descriptor data being the most common. The sweetpotato collection at CIP, which started later than potato, has over 3400 accessions with some associated data yet, some traits are only evaluated for less than 10 accessions.

Many accessions maintained in genebanks were originally collected as populations and not as individuals, meaning the genotype and phenotype within an accession can be vastly different from plant to plant; therefore, purification of seed lots that are heterogeneous needs to be a priority to make these accessions of value to breeders or for molecular/genomic analyses. Once a seed lot is purified then specific traits can be tagged to an accession and made available to others. The major limitation with this philosophy is that if you purify each accession with an average of 10 different phenotypes then you increase the size of the collection 10-fold and if you multiple this by thousands of accessions, the number of accessions to maintain becomes overwhelming for genebanks that are already limited in terms of resources. Also, some crops are not amenable to selfing, and thus, cannot be easily purified so clonal maintenance is the only solution to maintain specific genotypes. Maintaining clones is considerably more expensive than seed collections.

In addition to identifying and purifying heterogenous accessions, prebreeding may be required to produce a line worthy of future crosses for a breeder. Prebreeding identifies and captures desirable characteristics from unadapted plants that cannot be directly used in breeding populations and transfers these genes into an intermediate stage that can be directly used by a breeder. This is necessary for accessions not adapted to a particular target environment, closely related wild species that can cross to the cultivated form, and for distant wild species that are difficult to cross.^9^ Some curators of genebank crop collections have a background in breeding and therefore can facilitate prebreeding work to aid crop breeders and provide improved material. Many genebanks, however, do not have dedicated prebreeders, and thus, scientists outside of the genebank or individual breeders carry out this work independently, which usually means the information gained is not deposited back to the public genebank dbase. In addition, the information obtained is often decoupled from the original accession because the focus for phenotyping is on the new progeny or progenies produced from a cross and not the original accession from the genebank. Although genebanks always encourage feedback of data from all users of genetic resources, seldom does data return to the genebank to add value back to the original accession.

Curators do regularly measure morphological descriptors on the accessions they maintain and regenerate, which in effect describe particular attributes of the accession such as flower color, leaf shape, and so on; however, these traits are largely ignored^3^ and often are of little value to breeders. Generally, the morphological descriptors selected for characterizing genebank accessions are chosen based on characters that do not vary dramatically between environments yet have a strong genetic component so that one accession can be differentiated from another over several environments. Characterization is normally done in very irregular cycles over multiple years as the accession needs regenerating due to low viability, seed stocks become low, and with different sets of accessions in each regeneration cycle. Next generation phenotyping and genotyping have emerged as methods to capture quality data on genetic resources, but these techniques are often quite costly for a genebank to procure and require considerable IT infrastructure to implement these systems.^3,4^ For genebanks to stay relevant in the 21st century, it is necessary to embrace the digital information age and invest in the infrastructure to provide useful data (genomics and phenomics) to its users.

To promote and enhance germplasm use, value needs to be added in the form of comprehensive cataloging and association of important traits to each accession. Users of genetic resources are interested in various traits, though yield quality traits, nutrients, and disease resistance are among the most commonly requested. There are several methods that can be utilized to link or discover traits in accessions, such as standard phenotyping, marker assisted selection (MAS), genome wide association studies (GWAS), prediction based on known data, core/mini core collections, and mining publications and public datasets for information. These various methods along with a few select examples of each method are discussed in this review (Table 1). This is not meant to be an exhaustive list of all methods and examples of each method, but an overview of some useful methods to link traits to accessions.

**Table 1.** Different Approaches Discussed in This Review That Genebanks Can Utilize to Link Traits to Accessions Along with Their Major Advantages and Disadvantages

*Methods*	*Advantages*	*Disadvantages*
Mining public data	Low cost, no research required, only cost is personnel time to mine data and format information.	Lose quality control, no input on experimental design, may not include enough replications or GXE analysis, difficult to summarize all meta data within genebank dbase or harmonize among scoring of traits from different experiments/labs.
User feedback	No cost, long-term users in the community are invested and are conscientious about data fidelity/quality.	Difficult to receive feedback before publication and after publication no feedback is generally ever received, need more than anecdotal evidence to link trait to accession.
Brute force phenotyping	Quality control, traits important to the breeding/user community can be selected.	Requires significant funding and personnel time especially for large numbers of accessions and multi-location testing.
Core/Mini core	Reduces number of accessions to screen for a trait of interest.	Not all desired traits can be found in a core/mini core.
Focused identification of germplasm strategy	Limits number of accessions to screen and gives a “best bet” of accessions to find trait of interest using available data. High probability of finding sought traits in a manageable subset.	Traits are not always predictable based on the information available and complexity of the trait. Some evaluation data needed to develop the algorithms for predicting the relationship between the environmental conditions and the trait.
Marker assisted selection	Straightforward approach to identify traits without needing mature plants to evaluate, can screen large numbers of accessions efficiently.	Does not work on complex quantitative traits, expensive if markers are not already developed, requires specialized laboratory equipment.
Genome wide association studies	Links markers to traits of interest that can be used for selection/screening in germplasm, traits with few loci with large effects work well. Families not required.	Spurious associations occur so validation is required, genotyping and phenotyping is required so cost is significant, complex traits can be difficult for GWAS, no guarantee on which trait(s) will have strong association to molecular markers. Affected by heritability, GxE, population size, and genotyping quality.

GWAS, genome wide association studies; GxE, genotype by environment.

## Methods

One fairly easy, low cost way to link traits to accessions is by mining publicly available data. The only requirement is staff time needed to search for the information and format it appropriately. Searches can be made for the crop of interest to discover publications and evaluate the samples chosen in each study that were distributed from the genebank. Open access datasets that have now become a fairly standard requirement can be mined to find genebank accessions. In the case of CGIAR (Consultative Group on International Agricultural Research) datasets and publications, dataverse^*^ and CGspace^**^, respectively are the current mandatory data repositories. Scientific journals are also now requiring depositing of data sets in supplemental links to a published article. Once potential publications or datasets are identified with genebank accessions, the trait data collected can be summarized and tagged to an accession. In the last 2 years, the International Potato Center (CIP) genebank has been using this method to expand the available trait information. This method has provided data collected by other researchers on 26 traits for 2995 accessions from the genebank of which 529 and 2466 were sweetpotato and potato, respectively. Information collected for potato included traits for resistance to late blight and bacterial wilt, vitamin C content, anthocyanin, dry matter, tuber bulking maturity, sugar content, glycoalkaloids, cooking and post cooking quality measurements, chipping color, and drought index. In sweetpotato, traits such as drought tolerance, sweetpotato virus disease resistance, yield, β-carotene, dry matter, total sugars, starch, and protein were associated with accessions to aid in germplasm selection. Overall, this has proven to be a low-cost method of gaining information from previously conducted studies on genebank accessions.

Another potential strategy is to request and encourage researchers and breeders to provide data back to the genebank from material they have requested and evaluated. This can be successful and genebanks receive the information usually in the form of a publication and can make the data publicly available without the expense of having to collect the data. In reality, most of the time, no information is returned even when stakeholders request the germplasm to screen for a trait of interest.^3^ Breeders are generally willing to support the genebank with trait information on accessions; however, the main limitation is that breeders request material, make a cross, and then characterize the progeny/derivatives of genebank accessions with the resulting information pertaining to a new genotype which is not easily linked to the original genebank accession requested.

### Brute force

Another straight forward way to link traits to germplasm accessions is to put the effort into phenotyping for a trait of interest. This approach often requires considerable effort from staff, multiple years of planning, and separate financial support to accomplish. Funding to genebanks usually only covers the basic maintenance of the collection and not evaluation work, and several studies have demonstrated that the majority of genebanks lack sufficient funds to cover basic facilities and staff to maintain their collections.^3^ Thus, evaluation of genebank collections is often only a feasible strategy for smaller genetic resource collections containing a few dozen to a few hundred accessions. The biggest limitations, therefore, are resources and balancing the goals for maintaining collections with the number of available staff, financial support, number of accessions that can be evaluated, and a reasonable time period to complete a project. Further, a trait needs to be fairly easy and inexpensive to measure without destroying a lot of the plant material, otherwise considerable effort will also be needed to regenerate the accession(s).

In this approach, the evaluation needs to be mainly restricted to agronomic traits showing high heritability.^10^ This is because plants display phenotypic plasticity where one genotype can produce multiple phenotypes due to the environmental conditions such as response to shade or light, architectural changes above ground due to nutrients or changes to root structure in differing soil types affecting access to water.^11^ This further reinforces that evaluation data needs to be collected at multiple locations to understand the level of plasticity for a particular trait. Because traits can be influenced by the environment, it is critical that GxE is addressed by collecting trait data in multiple environments. While evaluation of a trait(s) in a single environment over a single year does provide some baseline information on the range of variation among different genotypes, it does not provide any information on how that accession may respond in an environment different to the one in which it was evaluated.

One example of phenotyping an entire collection by brute force is from the USDA castor bean germplasm collection (1033 accessions) that was measured for total oil content and fatty acid composition.^12^ Castor seeds contain a toxin and are unsafe for consumption; however, the oil is edible, highly viscous, and is used in various cosmetics and lubricants for high speed engines. Total oil content was measured by nuclear magnetic resonance (NMR), which is a nondestructive approach and does not require many seeds. The variation in the collection ranged from 37.2% to 60.6% oil content. Unlike NMR, gas chromatography (GC) is a destructive process, but it is fairly cost efficient, can be automated, and requires only part of a single seed up to a few seeds for analysis. The GC analysis of the castor bean collection demonstrated significant variability for the following fatty acids: ricinoleic acid (C18:1-1OH), linoleic (C18:2), oleic (C18:1), and stearic (C18:0), while the range of variability for the remaining fatty acids was rather small.^12^ The evaluation information collected in such studies is a long-term resource for breeders and researchers to select ideal germplasm for improving total oil or fatty acid profiles in castor bean.

Cucumber is an old crop that has been cultivated for 5000 years and grown throughout the world for the fresh or processed vegetable market.^13^ The entire USDA cucumber germplasm collection was evaluated for three years for fruit yield and quality traits. Interestingly, the environment did not play a significant role for any of the traits evaluated. Accessions with the highest fruit number were identified and some had higher yields than the checks included in the study.^13^ All of the data from this study was made available on GRIN^†^, making it easy for users to evaluate this data and make selections for their breeding or research programs.

Phenotyping an entire collection is noteworthy, although this is not always feasible due to the cost involved for large genetic resources collections. Therefore, stratification strategies can be applied to choose a manageable number of accessions with knowledge gained on a select set by phenotyping a portion of the collection. One example of this is resistance to rice blast that causes significant yield losses in this major cereal crop.^14^ In rice blast, it is critical to look for multiple forms of resistance because the causal agent (fungi) rapidly overcomes any single form of resistance after only a few years of agricultural use; therefore, continuous searches are ongoing for new germplasm containing resistance. A total of 4246 accessions were screened from the International Rice Research Institute (IRRI) genebank and 74.8% of these accessions were found to be resistant. However, only 289 genotypes (7%) showed resistance to all five rice blast isolates.^14^

In another study^15^ over half (55%) of the watermelon genebank from the USDA, which included three different species and material originating from 57 different countries, were examined for tolerance to drought stress, an important trait due to the growing threat of climate change. This screen demonstrated that the most drought tolerant material originated from desert regions in Africa. Of these, two identified drought tolerant accessions also had resistance to papaya ringspot type-W and zucchini yellow mosaic virus.^15–17^ These accessions containing drought and virus resistance are ideal candidates as parents in a breeding program for stacking multiple traits.

Oil of palm is an important source of edible oil; however, these oils can oxidize, which affects the overall quality of the oil. If endogenous enzymes (lipase) are reduced in a particular genotype, then the shelf life of a product is improved and lengthened. Palms from the Malaysian Palm Genebank (148 accessions) were screened for lipase activity. Low and high lipase materials were identified and found to be correlated with geographic origin with low lipase palms coming from countries bordering the Sahara desert and high lipase palms derived from areas with higher rainfall, which was consistent with the biology in which the enzyme needs water to hydrolyze the oil.^18^

Huanglongbing (HLB) is a destructive disease to the citrus industry that has spread in the primary growing areas of the United States since the mid 2000s. The focus in the citrus world is to find resistant and/or tolerant cultivars. Eighty-three accessions representing 85% of the genetic diversity of citrus and its wild relatives were evaluated under field conditions to determine tolerance to HLB. Of the accessions evaluated, the best performing ones under HLB pressure included citrons (*Citrus medica*), accessions with citron pedigrees, and the wild relatives all of which had low or no symptoms of HLB.^19^ These accessions can be candidate accessions used by the breeders to develop more tolerant cultivars.

## Core and Mini Core Collections

The concept of core collections was originally proposed by Brown.^20^ The idea is to make a subset of a germplasm collection that consists of the majority of genetic variation with little genetic redundancy since it is generally not possible for a researcher to screen every accession within a genebank for a particular trait(s). Data such as geographic origin, specific plant characteristics, trait data, and molecular data are utilized to develop core subsets. Because a core is a much smaller subset, it facilitates evaluation and characterization more efficiently and effectively.^6^ The core collection concept allows researchers to screen a smaller set of samples, generally 10% of the total collection that approximately captures 70% of the total genetic diversity, to save time and resources to find the trait(s) of interest. Certainly this is not the case with crops such as maize, wheat, and rice with extremely large holdings >25,000–120,000 accessions, where a 10% set will still be a very large number of accessions to screen. In these cases, a core collection would generally be too large to evaluate at multiple locations with replication^21^ without extensive resources.

There are many methods available and free software packages such as MSTRAT,^22^ PowerCore,^23^ ccChooser,^24^ CoreHunter,^25^ and GenoCore^26^ among others that now are available to help construct a core or mini core collection. Many of these programs can use molecular marker data, genetic distances, phenotypic traits, geographic origin, or integration of these various data types to select a core set.

A further point of consideration is that core collections should be dynamic, not static. A periodic review and modification of the core collection may be warranted as genebank holdings increase and new diversity is added or when new information on accessions becomes available. As an example, the core subset in yam was revised from 371 to 843 accessions because the International Institute of Tropical Agriculture (IITA) genebank had increased its collection over time by acquiring material from Benin and Togo, and new information that had been collected on duplicates within the collection and sorting of genetic identity issues.^27^ Additionally, the pearl millet core collection from the International Crops Research Institute for Semi-Arid Tropics (ICRISAT) was modified to add 501 accessions from accessions that were characterized after the original construction of the initial core set.^28^

Core collections have been constructed for numerous crops from many genebank collections worldwide. For example, core collections have been developed from the USDA chile pepper germplasm,^29^ Universidad Nacional del Altiplano (UNAP) collection of quinoa,^30^ IITA germplasm collection of yam,^27,31^ USDA peanut collection,^32^ ICRISAT groundnut collection,^33^ Beijing Vegetable Research Center (BVRC) watermelon germplasm,^34^ ICRISAT chickpea germplasm,^35^ and the Worldwide Olive Germplasm bank (OWGB)^36^ to name a few. One element often missing, however, is a comparison of core collections between genebank collections that hold the same crop. A comparison was made between the peanut mini core from China and the ICRISAT mini core, which were both evaluated using simple sequence repeat (SSR) markers. The genetic distance between the two subsets was larger than the distance within a single mini core, suggesting that the material was fairly unique. Overall, the diversity was higher in the Chinese mini core than the ICRISAT mini core.^37^

Evaluations of these core collections from genetic resource collections has led to comprehensive cataloging of germplasm, and, important discoveries of traits that breeders can use to make selections for improving crops of interest. A core collection for the common bean was evaluated for trace minerals,^38^ and genetic variability of iron and zinc concentrations ranged from 34 to 89 and 21 to 54 mg/kg, respectively. Tannins were also evaluated in this study because high levels of tannins tend to reduce the availability of iron levels in food preparation or digestion. Colored seeds are often associated with higher tannin levels, but this study demonstrated that colored seeds had a large range of variation, suggesting it is possible to reduce tannin levels even for the darker colored seeds.^38^

In wheat, a core collection of 372 accessions from the Clermont-Ferrand Genetic Resources Center, France, was chosen based on passport and microsatellite data. Various agronomic and quality traits in the core were evaluated and compared to modern varieties to assess the diversity within the core subset.^39^ The wheat core from this collection had a large range in protein content (10.9%–19.2%), which is important for determining the nutritional value. Preharvest sprouting ranged from 0% to 61.3% and the quality of the wheat for bread making ranged from tough, inelastic dough to high quality dough for bread. Bordes et al.^39^ found that the modern varieties (1960–2000) in the core collection typically had a smaller range of variation than the landrace or older varieties for several of the traits evaluated in the core. This comprehensive evaluation of wheat allows breeders to select accessions for breeding programs with the traits they desire.

While core collections can often lead to the identification of accessions with specific traits needed by breeders for crop improvement, sometimes this strategy does not work to identify a trait of interest. Food allergens are a significant problem around the world. In soybeans, the seeds are a major source of human allergens (i.e-P34-cysteine protease). Soy is used in processed foods making those with food allergies vigilant in checking all the ingredients from any of the products they consume. The soybean core collection and a group of wild relatives were evaluated for P34 and other seed allergens.^40^ All of the core lines and other accessions assayed showed the presence of P34, indicating that this protein is highly conserved in soybean, which suggests breeding to eliminate this major allergen will be difficult.^40^ These results further suggest that without genetic modification to knock out the gene(s) involved in P34 synthesis, there likely will be no solution to obtaining a nonallergenic soybean.

The mini core concept was proposed in the early 2000s^41^ because for screening of certain traits, a core collection is still too large. A mini core is ∼1% of the entire germplasm collection and derived from accessions within a core collection and is thus a subsample of the core collection. The advantage of the core and mini core strategy is that once accessions are found with a particular trait of interest, a user can back track to the clusters these accessions originally were grouped in and screen more accessions from that particular cluster to find additional individuals with a trait of interest. Because accessions are grouped together based on some commonality, in general the accessions in the germplasm collection that were not selected for inclusion in the core, but reside in the same cluster as the accession identified with a unique trait, often may contain the trait of interest. This particular method has been successful in identifying additional accessions from a collection with the desired trait(s).

Diseases are one of the main limitations to yield potential in all crop plants, thus, identifying new sources of resistance that can be used in breeding programs is always needed. Mini cores have been an effective tool to mine germplasm for needed resistant accessions. Grain mold and downy mildew are diseases that affect the yield of sorghum, an important cereal crop worldwide. A mini core collection was screened for resistance to these diseases and a total of 50 accessions were identified as resistant to grain mold and six accessions were resistant to downy mildew from the ICRISAT genebank collection. One accession was identified that was resistant both to downy mildew and grain mold.^42^ In chickpea, fungal diseases hamper yield potential of this important pulse crop. To find resistant material for breeding programs, the mini core collection (211 accessions) was screened for resistance to several fungal diseases under controlled conditions. None of the accessions were found to be resistant to the multiple diseases screened. However, 25 accessions were resistant to Fusarium wilt, three moderately resistant to Ascochyta blight, 55 moderately resistant to Botrytis gray mold, and six accessions to dry root rot.^43^ Fusarium wilt and sterility mosaic disease affect pigeonpea production. The pigeonpea mini core subset (146 accessions) was found to contain six accessions with resistance to Fusarium wilt and 24 accessions with resistance to sterility mosaic disease.^44^

## Focused Identification of Germplasm Strategy: Predicting Traits via Machine Learning

The Focused Identification of Germplasm Strategy (FIGS) has been developed jointly by the International Center for Agricultural Research in the Dry Areas (ICARDA) with the Vavilov Institute (Russia) and the Australian Winter Cereals Collection as an approach to address utilization of genetic resources. The premise behind the FIGS approach is that the environment under which wild and landrace material grows will drive the evolution and selection of adaptive traits that could be of use to plant breeders. The method seeks to determine a potential relationship between collection site (agro-climatic conditions) and the presence of specific traits, such as resistance to biotic stresses and tolerance to abiotic stresses. The process identifies candidate collection sites that are likely to have imposed selection pressure for the trait of interest, which in turn allows the germplasm curator to identify a best-bet set of germplasm for evaluation.^45^ This approach is supposed to provide a better alternative to random sampling and use of core collections since it is specific to each trait and is selecting manageable size subsets with higher probability of finding the desired traits. FIGS has demonstrated its relevance and efficiency in identifying specific traits for breeders rapidly and precisely. In recent years, it has allowed the identification of new allelic variation and novel genes for traits that researchers have been looking for, unsuccessfully, for a number of years (Table 2).

**Table 2.** Confirmed Traits Identified by the Focused Identification of Germplasm Strategy Approach for Wheat, Barley, and Faba Bean

*Trait*	*Crop*	*References*
Resistance to Russian Wheat Aphid	Wheat	^74^
Resistance to stem rust (UG99)	Wheat	^75,76^
Resistance to yellow or stripe rust	Wheat	^77^
Resistance to powdery mildew	Wheat	^64^
Resistance to net blotch	Barley	^78^
Resistance to Sunn pest	Wheat	^79^
Tolerance to drought	Faba bean	^80^
Tolerance to boron toxicity	Wheat	^45^
Water use efficiency	Faba bean	^80^

During the last 10 years, ICARDA, with the financial support of GRDC/Australia, is taking the lead to further develop and improve the FIGS pathways through testing different modeling processes and generating long-term layers for main climatic variables and onset layers for ICARDA main crops (wheat, barley, chickpea, lentil, faba bean, and grass pea). Fine-tuning the FIGS approach is a continuous process since it is model based. ICARDA and its collaborators are evaluating FIGS subsets and generating feedback to improve the models and algorithms used for sub-setting. This improvement is also enriched by access to evaluation data around the globe. Furthermore, the availability of molecular data will be an added-value for FIGS processing by integrating marker-trait association and maximizing genetic diversity in the selection of trait subsets. Environmental data is a limiting factor in FIGS processing and more precision in daily climatic data for crops is needed in order that effective modeling can occur to evaluate traits that are influenced by specific growing periods. Another limiting factor is the lack of information of virulence spectra of pests.

FIGS follows two distinct pathways: filtering and modeling, both of which select best-bet environments that are likely to have imposed selection pressure for specific traits on *in situ* populations over time. Developing a FIGS filtering strategy requires deep understanding of the ecology and the optimal conditions of the expression of the trait under study, how these conditions affect the crop, and how this will relate to a selection pressure on an *in situ* population. Filters can be applied in the search process, such as excluding regions where a particular disease has not been reported or restricting a search to collection sites where stresses have occurred. Since most biotic and abiotic stresses happen at a very specific growing stage(s), daily long-term data together with the crop onset information are required to zoom into the crop growing stages using growing degree days.

When evaluation data is available or a user has a clear idea about the classification of an adaptive trait based on knowledge such as heat, drought, or frost, FIGS can explore the mathematical relationship between the adaptive trait of interest and the long-term climatic and/or soil characteristics of collection sites. The mathematical conceptual framework of FIGS is based on the paradigm that the trait as a response variable (*Y*) depends on the environment (*X*), where *X* = (*x*_1_, … , *x_n_*) are the covariates. The quantification process leads to the generation of *a priori* information, which is used in the prediction of accessions that would carry the desired trait.

The performance of the models/classifiers is measured using the metric parameters derived from a confusion or error matrix, an *n* by *n* (*n* number of classes in the trait) matrix presenting the percentage of true positives (TP), false positives (FP), false negatives (FN), and true negatives (TN). Accuracy (how often the classification is right), Kappa (accuracy vs. random chance), sensitivity (proportion of truly positives cases), and specificity (proportion of truly negative cases) are the metrics used in addition to the area under the curve to discriminate between different models and assess the trait/environment association. The high accuracy of the models is an indication of the presence of the trait environment association. This information is used in predicting accessions that could carry the trait at a higher frequency than a random selection of accessions.

FIGS uses several machine learning techniques including nearest neighbors k-nearest neighbors (kNN),^46^ support vector machine (SVM),^47^ and random forest (RF).^48^ R language^49^ is used as an open source platform for FIGS development and is the most appropriate for packaging FIGS steps to conduct research and to communicate the results to the global plant genetics community. The first version of the R-FIGS package will be published in GitHub and will be available in 2018.

### Case study and predictive characterization using FIGS

**FIG. 1.**
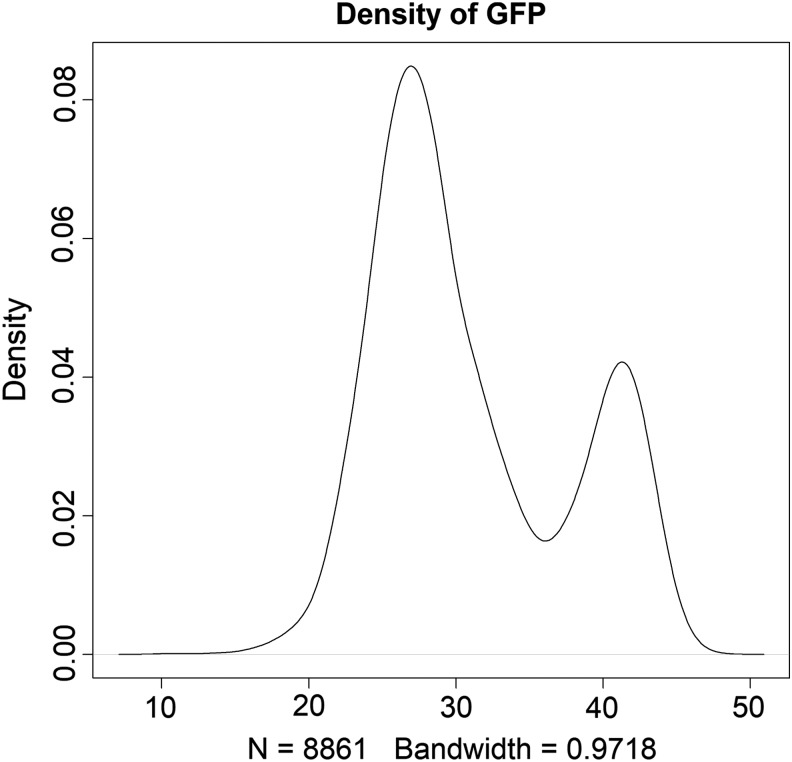
Density of GFP using 8861 data points. A biomodal distribution was produced from the information in the genebank. The *x*-asis is GFP in days and the *y*-axis is the density. GFP, grain filling period.

**FIG. 2.**
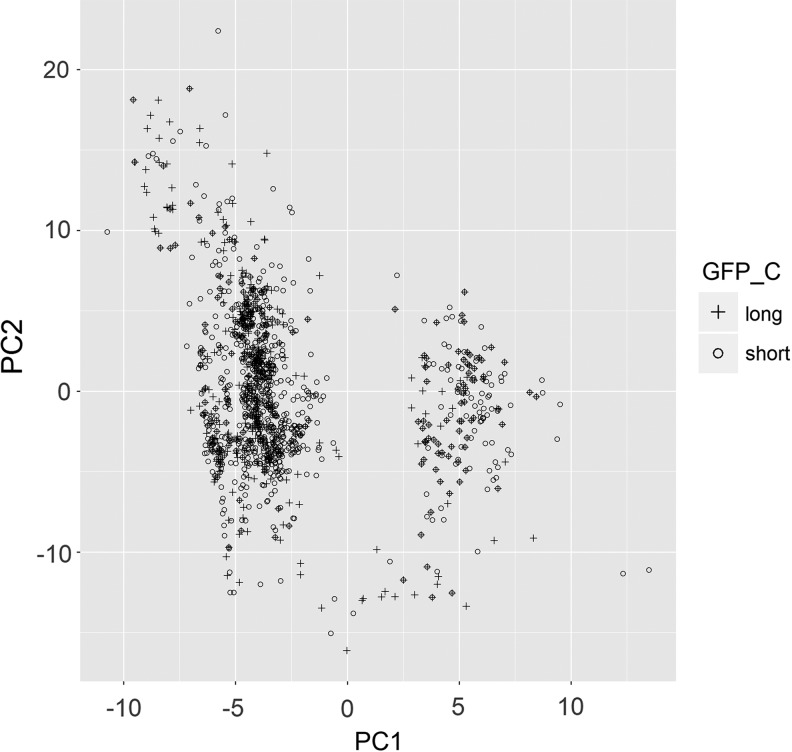
Scatter plot (PC1 vs. PC2) of landraces resulting from Principle Components Analysis using climatic data. The *dots* symbolize the landrace's GFP class (*plus* for long GFP and *circles* for short GFP).

The ICARDA genebank holds around 14,800 georeferenced durum wheat landraces, of which ∼9000 were evaluated for phenology. The grain filling period (GFP) was then estimated as the difference between days to maturity and days to heading. The evaluation was done at the ICARDA station TelHadya in Syria during the 1991 growing season. The distribution of the GFP trait shows a bimodal distribution (Fig. 1) for the ICARDA durum wheat landraces and validates that the ICARDA durum wheat landraces can be classified as having short (6486 accessions) or long GFPs (2375 accessions). The first two components from the principal component analysis using WorldClim data^50,51^—explained more than 80% of the total climatic variation, but failed at classifying the durum landraces into short or long GFPs (Fig. 2). Climatic information together with multivariate statistical techniques did not classify the ICARDA durum wheat landraces regarding GFP characterization.

**FIG. 3.**
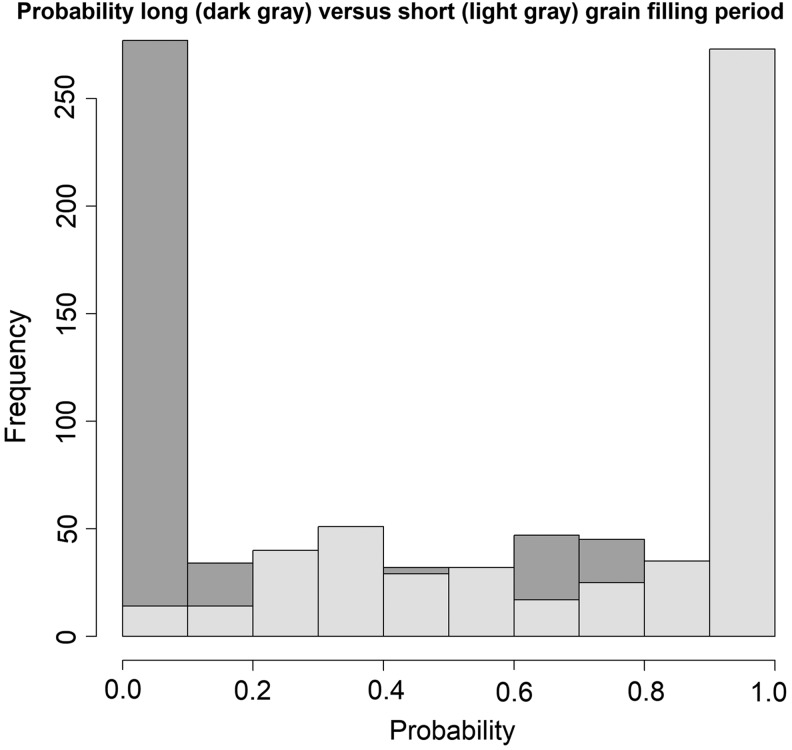
Predictive probability for the entire ICARDA durum wheat landrace collection based on machine learning model. *Dark* and *light gray* are the probabilities of being classified as long or short GFP, respectively. ICARDA, International Center for Agricultural Research in the Dry Areas.

**FIG. 4.**
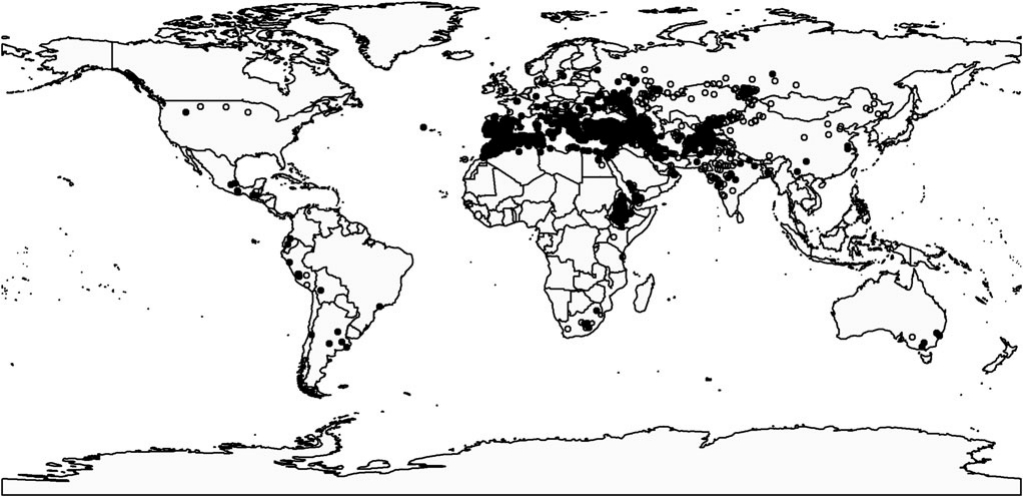
Predictive GFP class for the entire ICARDA durum wheat landrace collection, *white* and *black circles* are long and short GFP landraces, respectively.

The data was subsequently split into two sets: training (75%) and validation (25%). Three machine learning classification algorithms kNN, RF, and SVM were run. First the algorithms were tuned (adjustments to the model) to choose the best parameters for each algorithm, tree numbers and the number of predictors (mtry for RF), *k* number of neighbors for kNN, and *C* and gamma for SVM. Finally, the three models were trained using the training set with the optimal tuning parameters and extraction of the metrics. RF was the most accurate model with good Kappa, sensitivity, and specificity values (Table 3). High metrics (Table 3) validated that there is an association between WorldClim data and GFP for the evaluated durum wheat set. The constructed model was then used to predict the entire ICARDA durum collection with a probability of being short or long GFP (Figs. 3 and 4). The FIGS model can therefore be used as a predictive characterization technique in the sense that a probability of a trait's presence is assigned to uncharacterized germplasm in an *ex situ* collection.

**Table 3.** Performance Metrics for Three Machine Learning Classification Algorithms

*Performance measures*	*k-Nearest neighbors (kNN)*	*Random forest (RF)*	*Support vector machine (SVM)*
Accuracy	0.834	0.838	0.817
95% CI	0.799–0.865	0.804–0.868	0.781–0.849
No information rate	0.762	0.762	0.762
*p*-Value (Acc>NIR)	3.58E-05	1.37E-05	0.001423371
Kappa	0.563	0.557	0.467
Sensitivity	0.722	0.675	0.54
Specificity	0.869	0.889	0.903

Accuracy is the fraction of predictions our model got right, 95% CI: confidence interval for accuracy, Kappa compares an observed accuracy with an expected accuracy (random chance), sensitivity—the proportion of truly positives cases that were classified as positive, specificity is the proportion of truly negative cases that were classified as negative, and NIR is the proportion of the data with the majority class and a *p*-value to test that accuracy is better than NIR.

CI, confidence interval; NIR, no information rate.

The focused identification of the germplasm strategy is a powerful tool aiming to reduce the number of accessions that breeders need to screen and maximizing the chance of finding novel alleles for the targeted adaptive trait in a predicted subset. Similar approaches are also being investigated for searching useful traits *in situ* to guide future collecting missions. The ultimate goal is to develop a friendly package in R that could be available to users worldwide for efficient mining of genebank collections.

## Molecular Methods

MAS has been utilized as a means to gain valuable information in segregating populations to rapidly identify undesirable genotypes or to classify a set of genebank accessions for a trait of interest. Utilization of markers saves a lot of valuable time by identifying material with a particular trait of interest at an early stage of development rather than waiting for full maturity of a plant to be able to phenotype for that trait. This approach is especially useful in crops with long periods of juvenility. If a marker is tightly linked to a trait or if it is designed to detect a functional mutation within a gene, accessions can be interrogated with the marker(s) and the trait can be inferred and/or validated by phenotyping. The main limitation to MAS is it is only an effective tool when the trait is controlled by one or two genes or if the trait is under the control of few quantitative trait loci (QTLs) with large contributions to phenotypic variation.^52^ Further, MAS is only effective with major QTLs that have limited environmental or epistatic interactions.^53^ Lastly, the markers need to be highly reliable and reproducible among various labs and populations for this approach to be consistently successful.

The high oleic trait in peanut is an important seed quality trait. This trait gives peanut seed longer shelf stability (prevention of the oils going rancid) that is desired by manufacturers and provides the consumer with the health benefit of more monounsaturated fat in their diet with a fatty acid profile similar in composition to olive oil. Previous work has shown that two functional mutations G448A in *ahFAD2A* and 442insA in *ahFAD2B* were necessary to produce a high oleic peanut. Both of these mutations are required in the homozygous recessive state to significantly affect the function of the enzymes that convert oleic acid (18:1 monounsaturated fatty acid) to linoleic acid (18:2 polyunsaturated acid).^54–57^ Markers were developed to track these important mutations and the underlying trait^58,59^ in germplasm. Ninety-four accessions from the USDA mini core peanut collection were evaluated with these markers^60^ showing that the *ahFAD2A* mutation naturally existed in a homozygous state in 41% of the population whereas the *ahFAD2B* functional mutation was not detected.^61^ The alleles were also screened in 39 wild peanut species from the genebank to track the ancestry of these mutations; however, no functional mutations were detected in the wild accessions.^62^ Further, a study tracking the genotypes (*ahFAD2*) and the resulting phenotypes (fatty acid profiles) in segregating populations demonstrated that this trait was not controlled by dominant gene action as previously determined, but was quantitative in nature with much of the variability for three fatty acids (palmitic, oleic, and linoleic) being controlled by the two key genes (*ahFAD2A* and *ahFAD2B*), even though segregation patterns were typical of Mendelian inheritance from the two homoeologs. Another line of evidence of their quantitative nature was that several of the fatty acids were significantly positively and negatively correlated with one another.^63^

Where molecular markers can really expedite trait discovery is in crops with long periods of juvenility where years are required to produce the first fruits and/or significant land is needed for growing the crop. For instance, table grapes can take two to four years to produce fruit and then phenotyping would be required for several seasons after fruit production to evaluate a particular trait. Microsatellite markers linked to the seedlessness trait in grapes were employed to evaluate material in the *Vitis* germplasm bank from IMIDRA, Spain.^53^ Although the authors discuss the value of genotypic selection being superior over phenotypic selection, there were a few cases of false positives and negatives that were assumed to be caused by recombination between the marker and the gene, phenotypic misclassification, or a minor effect QTL. However, in most cases the markers were effective in the detection of the desired trait greatly speeding up the identification of seedlessness for breeders.

Another useful molecular approach in linking traits to genebank accessions is to sequence known functional genes from different individuals to identify the effect of different allelic variants. The most effective strategy for determining allelic richness is to sequence a collection of individuals to find the variants.^10^ FIGS was employed to define a subset of landrace accessions that were manageable for a molecular screening study in wheat.^64^ FIGS selected 1320 accessions from 323 different geographic origins that showed high selection pressure for powdery mildew resistance. These accessions were tested with isolates of powdery mildew and a total of 211 accessions showed complete or intermediate resistance to at least one race. The resistant accessions (56) were screened for the *Pm3* gene that is a known resistance gene and new allelic variants were identified by cloning and sequencing the gene from wheat accessions. Sequence data demonstrated 16 new allelic variants for the *Pm3* resistance gene. Bhullar et al.^64^ found that some of the resistant accessions had *Pm3* gene sequences identical to the susceptible alleles suggesting that there are more resistant genes in the genome to be discovered. Some of the new allelic variants identified were from accessions largely derived from Eastern Turkey. To verify whether these new allelic variants were linked to powdery mildew resistance, virus induced gene silencing VIGS was employed. This technique demonstrated that some of the new variants were indeed conferring the observed resistance and other variants either had another gene conferring resistance or the resistance was from a combination of *Pm3* and other genes. Overall, seven new *Pm3* alleles were described that represents a large allelic series of resistance genes with 14 allelic variants now described. Clearly, as demonstrated here, the diversity in genebank accessions can be utilized to identify important alleles from known resistance genes.^64^

## Genome Wide Association Studies

GWAS have emerged in the last 10 years as a powerful tool to link genetic markers to phenotypic variables in populations and further to discover genes and alleles for agricultural traits. It provides a connection between a trait and its underlying genetics. GWAS either identifies causative/predictive factors for a particular trait or it can provide information on the genetic architecture such as the number of loci and their contribution to the phenotype.^65^ This technique relies on linkage disequilibrium, which is nonrandom association of alleles in a population. The phenotypes collected for GWAS can be quantitative or qualitative. The potential for success in GWAS depends on the number of loci affecting the trait that are segregating in the population, allele frequency at these loci (genetic architecture), sample size, panel of markers used, and the heterogeneity of the trait.^66^ Further, finding an association between a genetic marker and a trait of interest is dependent on the variance of the phenotype in the population explained by that marker.^65^

GWAS and candidate gene sequencing-based association approaches were both utilized to evaluate marker trait associations in a chickpea reference set of 300 accessions derived from the ICRISAT genebank.^67^ Phenotyping was performed for 34 different traits under drought and heat stress over multiple years because drought can severely affect crop production. A combined approach of SSRs, diversity arrays technology, and single nucleotide polymorphisms (SNPs) were evaluated on the reference set along with sequence characterization of 10 drought related candidate genes, producing a total of 1872 markers. In total, 312 marker trait associations were found and 18 of the SNPs located in genes were significantly associated to the traits measured.^67^

In another study, GWAS was employed to locate SNP markers that are associated with variation in curd traits (edible inflorescence) in cauliflower, which is an important trait for yield.^68^ A total of 174 genebank accessions were evaluated for curd traits using over 120,000 SNP markers. A total of 24 SNPs were significantly associated with the curd traits.^68^ GWAS was also utilized to evaluate genotyping data produced from the “iCore” or informative core of 1860 barley accessions using three phenotypes deposited in the GRIN dbase.^69^ Significant SNPs were detected for the hull cover that were associated with the *NUD* locus and major genes determining spike row number.^69^

Soybean is an important source of oil and protein. However, salinization of land can affect soybean yields and phenotyping for salt tolerant lines in the greenhouse is expensive and time consuming whereas field selections can vary since salt concentration can range vastly in a particular field. Therefore, GWAS was employed using 33K SNP markers on a set of 283 accessions from the USDA Soybean germplasm collection. Soybeans from 29 different countries were utilized to avoid spurious associations from population structure and relatedness. Plants were treated with salt in the greenhouse. Chlorophyll concentrations were measured and chloride was extracted from the harvested dried leaves. This study demonstrated 45 SNPs from nine regions of the chromosomes associated with leaf chloride and leaf chlorophyll concentrations. Additionally, major QTLs associated with salt tolerance were also identified.^70^

Even though GWAS has brought about advancements in linking markers to traits for rapid selection, there are still some potential pitfalls. Complex traits are typically polygenic, and thus, have many loci contributing to the genetic variation observed; therefore, polymorphism in many genes play a part in the genetic variation observed in the population, so that the proportion of variance at the individual level is small.^66^ This means that individuals carry different alleles at multiple loci can increase and decrease the frequency or occurrence of the trait. In a population, there are many combinations of these alleles so that each individual can have a unique combination. Traits are often associated with variants at hundreds to thousands of loci and there is evidence of widespread pleiotropy for complex traits, implying that some variants affect more than one trait.^66^ Hence, large population sizes are needed for GWAS studies to determine the effect of each allelic combination on the particular trait being studied. Further, spurious signals can occur due to population structure or relatedness within the population. Signals determined from GWAS are often markers of putative risk and not the underlying functional genetic variant culprits, so caution should be taken before claiming variants have been identified.^71^ GWAS also does not provide information on the mechanism of how the genetic variant is associated with phenotypic differences or the target gene that controls the trait; however, new technologies are providing opportunities to bridge this knowledge gap.^66^ Another key point of all GWA studies is that after associations are identified, they need to be validated because there have been problems confirming the results.^72^ These associations also need to be generalized to other populations. Because a large amount of marker data is collected, a GWA study can identify numerous associations that are likely to be overestimates and unbiased effects can only be made in a data set not used in the discovery process.^73^ In summary, validation of GWAS results is an important factor to ensure linkage between a marker and a trait of interest.

## Conclusions

There are several ways in which important agronomic and quality traits can be linked to accessions. From predictive machine learning, molecular techniques such as MAS/GWAS, to brute force phenotyping, all of which can lead to uncovering the range of diversity in a set of accessions and reveal critical traits of interest for crop improvement. Once accessions with traits of interest are identified and made available, breeders can use this information to select parents for crossing and move forward on releasing new varieties to meet current needs. Overall, comprehensive characterization of genetic resources is critical to add value to accessions and to further help guide users on selection of appropriate germplasm for their specific needs.

## Notes

^*^ https://data.cipotato.org

^**^ https://cgspace.cgiar.org

^†^ www.ars-grin.gov
